# Blue-Light-Excited Eu^3+^/Sm^3+^ Co-Doped NaLa(MoO_4_)_2_ Phosphors: Synthesis, Characterizations and Red Emission Enhancement for WLEDs

**DOI:** 10.3390/ma11071090

**Published:** 2018-06-26

**Authors:** Man Yan, Guanghui Liu, Jiahao Wen, Yinlong Wang

**Affiliations:** 1 College of Materials Science and Engineering, Nanjing Tech University, Nanjing 210009, China; many230@njtech.edu.cn; 2 School of Resources, Environment and Materials, Guangxi University, Nanning 530004, China; 18477167778@163.com

**Keywords:** luminescence properties, molybdate, red-emitting phosphor, energy transfer

## Abstract

The system NaLa(MoO_4_)_2_:Eu^3+^/Sm^3+^ phosphors were prepared by solid-state reaction followed by heat treatment at 450–600 °C. As shown by X-ray powder diffraction, the phosphors had a body-centered tetragonal structure, high phase purity and high crystallinity. The photoluminescence measurements carried out under excitation at 464 nm indicated the main emission at about 615 nm corresponding to the electric dipole transition ^5^D_0_→^7^F_2_ of Eu^3+^, which agreed well with the emission wavelengths of the GaN-based blue LED chips. In NaLa(MoO_4_)_2_:Eu^3+^/Sm^3+^ phosphors, Sm^3+^ can efficiently transfer excitation energy to Eu^3+^, which resulted in a significant improvement of the fluorescence intensity, and the fluorescence intensity of the phosphors calcined at 550 °C maximized at the doping concentrations of Eu^3+^ and Sm^3+^ of 15.0 and 2.0 mol %, respectively. The decay curves and CIE (Commission Internationale de L’ Eclairage) coordinates of Sm^3+^ or/and Eu^3+^ doped phosphors were analyzed for the investigation of the energy transfer mechanism and color variation trend. Thus, NaLa(MoO_4_)_2_:Eu^3+^,Sm^3+^ can be classified as a potential red-emitting phosphor for white light-emitting diodes (WLEDs).

## 1. Introduction

These days, the white light-emitting diodes (WLEDs) regarded as the new-style solid-state photosources achieve much more research enthusiasm than before owing to the energy conservation and environmental protection, long service time, high reliability and function [1,2,3,4]. However, the development of WLEDs is largely limited because there is no excellent red phosphor [5,6]. In the last few years, great attention has been focused on double alkaline rare-earth molybdates in virtue of their unique structures and excellent luminous properties [7,8,9]. NaLa(MoO_4_)_2_ pertaining to the double molybdate family is of tetragonal structure with space group I4_1_/a wherein Mo^6+^ combining with four neighboring O^2−^ forms tetrahedral MoO_4_^2−^ anions to make phosphor matrix with high physical–chemical stability [10,11] and both Na^+^ and La^3+^ randomly occupy S_4_ lattice sites with no symmetrical center, which is propitious to relieve further parity forbidden of the electric dipole transition of RE^3+^ [12]. Furthermore, the MoO_4_^2^^−^ group can absorb the energy of the ultraviolet light through Mo-O charge transfer interaction, and then improve rare-earth fluorescence materials’ luminous efficiency and intensity via transferring energy to rare-earth ions [13,14].

Europium ion (Eu^3+^)-doped compounds are the most promising candidates as the efficient red phosphors for WLEDs due to their advantages of high color purity and the strongest characteristic red light emission peak at about 615 nm attributing to the ^5^D_0_→^7^F_2_ transition [15,16,17]. Nowadays, Eu^3+^-doped molybdates are regarded as excellent red phosphors, but possess low luminescence intensity, improvement of which has been tested through many efforts [18]. For example, Zuo et al. [5] found that red emission intensities of Eu^3+^ were overwhelmingly improved by co-doping Bi^3+^ in the KLa(MoO_4_)_2_ phosphors. Zhou et al. [19] reported that intensity of LiY(MoO_4_)_2_:Eu^3+^,Sm^3+^ phosphors was heightened when the compounds were prepared by sol–gel process. However, for all we know, no researchers have reported the energy transfer pathway or photoluminescence (PL) properties of the Sm^3+^/Eu^3+^ co-doped NaLa(MoO_4_)_2_ matrix.

Thus, we synthesized the Eu^3+^ or/and Sm^3+^ doped NaLa(MoO_4_)_2_ phosphors through a solid-state reaction. The influence of a calcination temperature on a crystal phase as well as luminescent intensity being discussed, and the photoluminescence (PL) spectra, decay behaviors and chromatic properties were analyzed at length.

## 2. Experimental

### 2.1. Synthesis

The Eu(NO_3_)_3_·6H_2_O (99.99%), Sm(NO_3_)_3_·6H_2_O (99.9%), La(NO_3_)_3_·6H_2_O (99.0%), NaNO_3_ (99.0%) and (NH_4_)_6_Mo_7_O_24_·4H_2_O (99.0%) used as raw materials were bought from Shanghai Aladdin Bio-Chem Technology Co., Ltd. (Shanghai, China). The Eu^3+^-doped NaLa(MoO_4_)_2_ (abbreviated as NL_1−x_MO:xEu^3+^; the mol % x = 5, 10, 15 and 20%) and Eu^3+^/Sm^3+^co-doped NaLa(MoO_4_)_2_ (abbreviated as NL_1__−x−y_MO:15%Eu^3+^,ySm^3+^; the mol % y = 0, 1, 2, 3, 4 and 5%) phosphors were prepared by solid-state reaction. The stoichiometric mixtures of NaNO_3_, La(NO_3_)_3_·6H_2_O, (NH_4_)_6_Mo_7_O_24_·4H_2_O, Eu(NO_3_)_3_/Sm(NO_3_)_3_ solution (1 M) were ground thoroughly for 1 h in a porcelain mortar to be uniform, and, then, they were oven-dried at 85 °C for 12 h. Finally, appropriate amounts of procedures were calcined in a muffle furnace at 450, 500, 550 or 600 °C for 360 min to form the final products.

### 2.2. Characterization

The phase composition of the products was determined by the X-ray diffraction (XRD) method (Rigaku-Dmax 2500 X-ray diffractometer ( Japanese science and science company, Tokyo, Japan) with graphite monochromatized Cu Kα radiation, scanning rate was 5°/min, 2θ = 10–75°). The photoluminescence excitation (PLE) and photoluminescence (PL) emission spectra were measured on a HORIBA Fluoromax-4 spectrophotometer (HORIBA Ltd, Irvine, CA, America) equipped with a 150 W Xe-lamp, and the fluorescent decay curves were determined by a Hamamatsu Quantaurus–Tau spectrophotometer (Japanese hamamon photonics Ltd, Hamamatsu, Japan) equipped with a 450 W xenon impulse lamp as the excitation source. The entire tests were put into effect under room temperature conditions.

## 3. Results and Discussion

### 3.1. XRD

**Figure 1:**
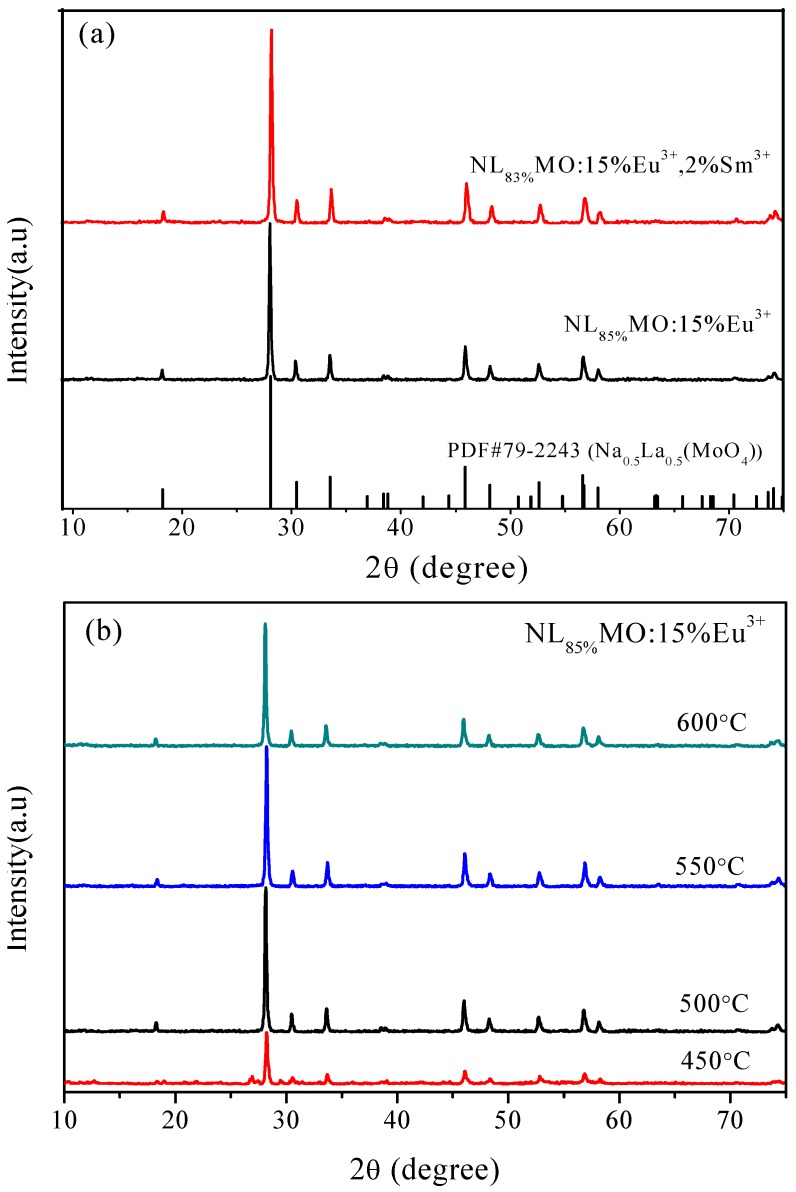
XRD patterns of (**a**) NL_83%_MO:15%Eu^3+^,2%Sm^3+^ and NL_85%_MO:15%Eu^3+^ obtained at 550 °C and (**b**) NL_85%_MO:15%Eu^3+^ formed at different temperatures for 6 h.

In Figure 1a, the two representative XRD patterns of NL_85%_MO:15%Eu^3+^ and NL_83%_MO:15%Eu^3+^,2%Sm^3+^ calcined at 550 °C for 6 h are shown. The diffraction peaks were well attributed to pure tetragonal phase NaLa(MoO_4_)_2_ (PDF Card 79-2243, a = b = 0.53424 nm, c = 1.17376 nm), indicating that the doped ions were successfully incorporated into matrix lattice and had not drastically altered the host structure. The presence of foreign phases was not detected. Figure 1b shows the XRD patterns of the products formed at 450, 500, 550 or 600 °C for 6 h. Obviously, the crystalline NLMO:Eu^3+^ has already formed at 450 °C and the sample calcined at 550 °C is better crystallized and possesses stronger and sharper peaks.

### 3.2. PL Properties of NLMO: Eu^3+^ Phosphors

**Figure 2:**
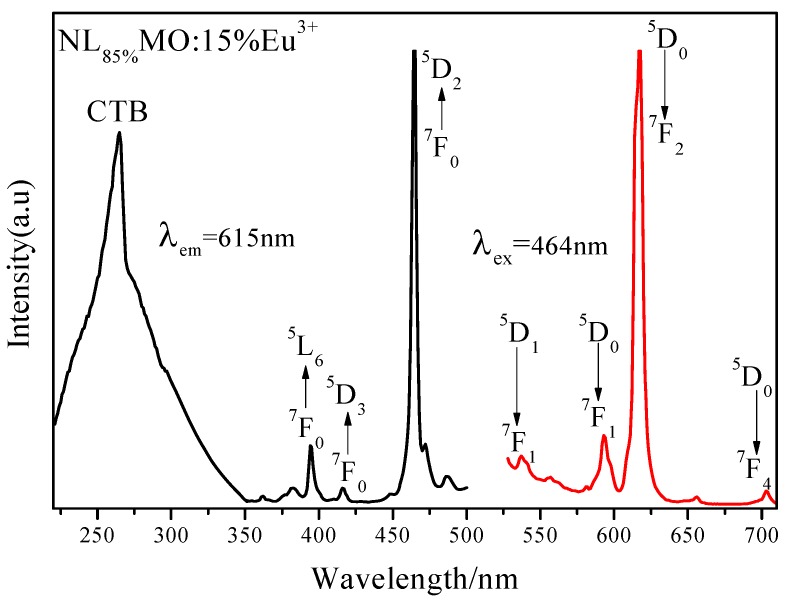
Excitation (λ_em_ = 615 nm) and emission (λ_ex_ = 464 nm) spectra of NL_85%_MO:15%Eu^3+^.

**Figure 3:**
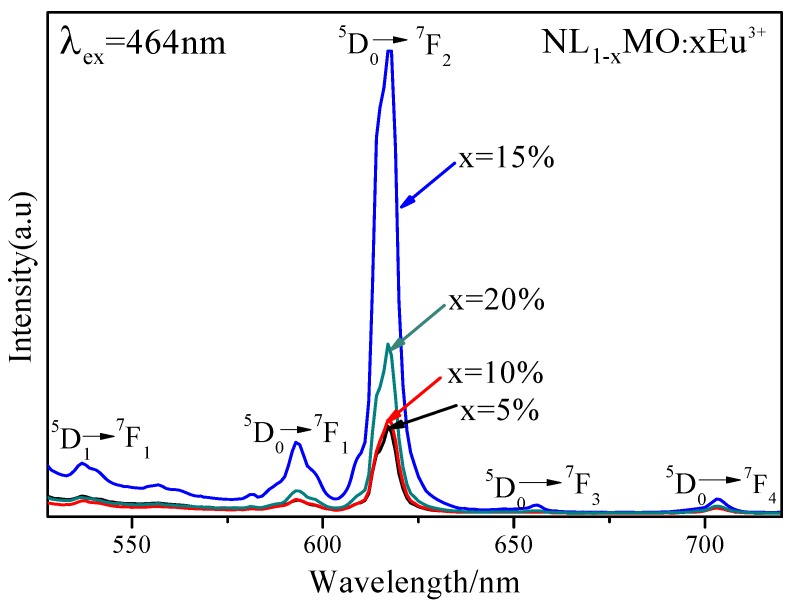
Emission (λ_ex_ = 464 nm) spectra of NaLa(MoO_4_)_2_ with Eu^3+^ concentration at 5, 10, 15 and 20 mol %.

**Figure 4:**
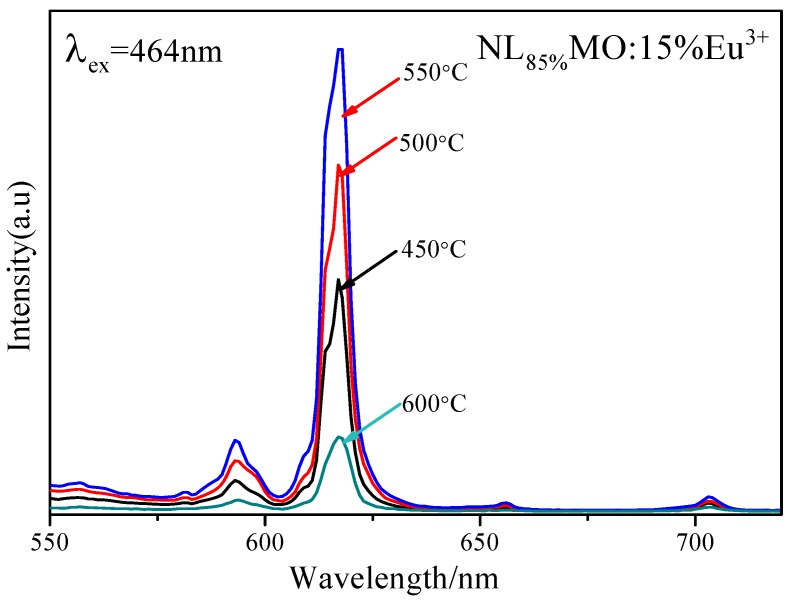
Emission (λ_ex_ = 464 nm) spectra of NL_85%_MO:15%Eu^3+^ obtained at different calcination temperatures.

It is generally known that Eu^3+^ as an excellent activator ion is extensively applied in commercial phosphors because its characteristic emission lines related to the ^5^D_0_→^7^F_J_ (J = 1–4) transitions are usually distributed in the red spectral region [20]. Clearly, in Figure 2, the PLE spectrum of NL_85%_MO:15%Eu^3+^ monitored with the emission at 615 nm correspondent to the ^5^D_0_→^7^F_2_ transition of Eu^3+^ is shown. There is a strong broad band ranging from 220 to 350 nm with a maximum at 265 nm as a result of the O-Mo and O-Eu charge transfers, as well as several sharp peaks within 350–500 nm. It is easy to ascribe the sharp peaks at 382, 394, 416 and 464 nm to the intra-configuration 4f–4f transitions of Eu^3+^ including ^7^F_0_→^5^G_J_,^5^L_7_, ^7^F_0_→^5^L_6_, ^7^F_0_→^5^D_3_ and ^7^F_0_→^5^D_2_, respectively. Among them, the strongest one at 464 nm is just agreed with the emission wavelengths of the GaN-based blue LED chips. The emission spectrum (PL) obtained under excitation of 464 nm consists of several sharp peaks at 537, 593, 615 and 703 nm due to the transitions of ^5^D_1_→^7^F_1_ and ^5^D_0_→^7^F_J_ (J = 1, 2, 4) of Eu^3+^, respectively. In particular, ^5^D_0_→^7^F_1_ belongs to magnetic and ^5^D_0_→^7^F_2_ to electric dipole transitions. According to the Judd–Ofelt theory [21,22], if Eu^3+^ is located at inversion symmetry, the ^5^D_0_→^7^F_1_ transition, which is scarcely affected by the crystal field environment of Eu^3+^, will play a leading role in the PL spectra and the phosphors emit an orange-red light when excited. Otherwise, the ^5^D_0_→^7^F_2_ transition is stronger and the phosphors emit a red light when excited. Moreover, the ^5^D_0_→^7^F_2_ transition is dominant in the PL spectra, which clearly indicates that Eu^3+^ occupies the La^3+^ site with anti-inversion symmetry and it means high red color purity. Here, the effects of Eu^3+^ concentration and synthesizing temperature on emission intensity are also shown in Figure 3 and Figure 4. Obviously, the optimal Eu^3+^ concentration and calcination temperature are 15 mol % and 550 °C, respectively, which were selected as the optimal conditions in the following experiments.

### 3.3. PL Properties and Energy Transfer Pathway of NLMO:Eu^3+^/Sm^3+^ Phosphors

**Figure 5:**
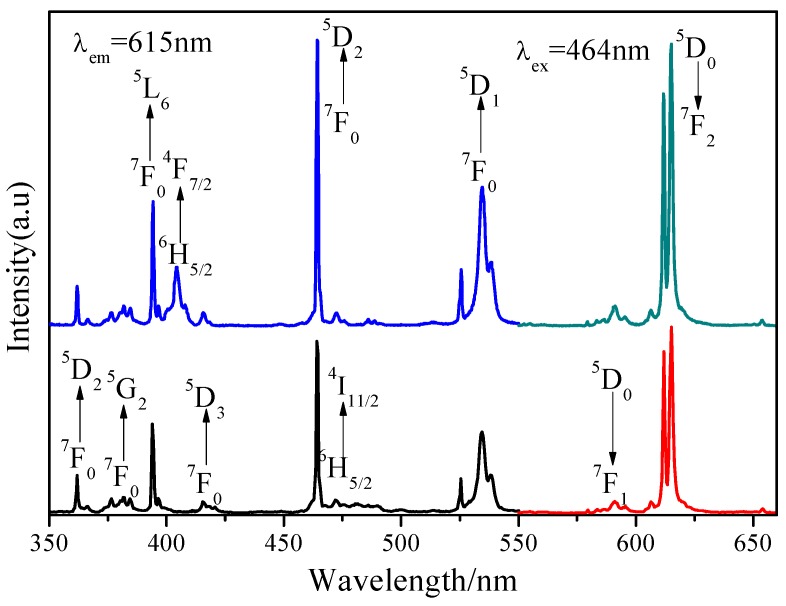
PLE and PL spectra of NL_85%_MO:15%Eu^3+^ and NL_83%_MO:15%Eu^3+^,2%Sm^3+^.

To investigate how Sm^3+^ transfers its energy to Eu^3+^ in NLMO, we analyzed the PLE and PL spectra in detail. Differing from the PLE spectrum of NL_85%_MO:15%Eu^3+^, that of NL_83%_MO:15%Eu^3+^,2%Sm^3+^ consists of the characteristic excitation peaks of Eu^3+^ at about 395, 464 and 535 nm and an excitation peak at about 403 nm attributed to the transition of ^6^H_5/2_→^4^F_7/2_ of Sm^3+^ (Figure 5).

**Figure 6:**
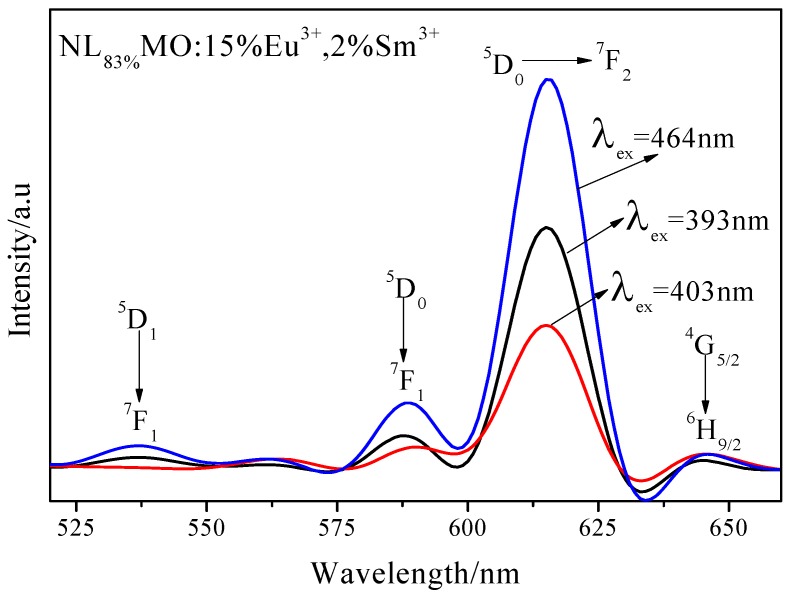
PL spectra of NL_83%_MO:15%Eu^3+^,2%Sm^3+^ under the excitation at 393,403 and 465 nm.

**Figure 7:**
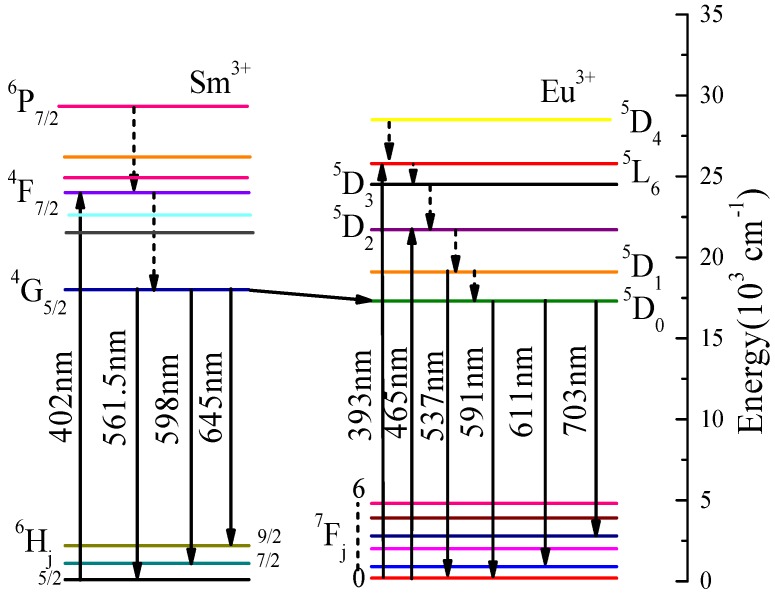
The energy transfer pathway between Eu^3+^ and Sm^3+^ in NaLa(MoO_4_)_2_.

The PL spectrum of NL_83%_MO:15%Eu^3+^,2%Sm^3+^ excited at 403 nm is closely similar to those excited at 393 and 465 nm, while the emission bands at 537 nm correspondent to the ^5^D_1_→^7^F_1_ transition of Eu^3+^ and the relative peak intensities are different (Figure 6). The three PL spectra exhibit two significant emission peaks at about 593 and 615 nm related to the transitions of ^5^D_0_→^7^F_1_ and ^5^D_0_→^7^F_2_ of Eu^3+^, respectively, indicating the Sm^3+^ transferred efficiently its excitation energy to Eu^3+^. Further comparison shows that there are the emission bands at 537 nm in NLMO:Eu^3+^ and NLMO:Eu^3+^/Sm^3+^ excited at 393 and 464 nm, but not in NLMO:Eu^3+^/Sm^3+^ when excited at 405 nm, while there are still other transitions of Eu^3+^. Therefore, the energy transfer just occurs from the ^4^G_5/2_ state of Sm^3+^ to the ^5^D_0_ state rather than the ^5^D_1_ state of Eu^3+^. The probable energy transfer pathway, as determined from the analysis above, is displayed in Figure 7. The electrons of Sm^3+^, after being excited into ^4^K_11/2_ level, rapidly relaxed down to the ^4^G_5/2_ state owing to lattice vibration, and then returned to the ground states through the energy transfer to the ^5^D_0_ level rather than the ^5^D_1_ level of Eu^3+^ [23,24].

**Figure 8:**
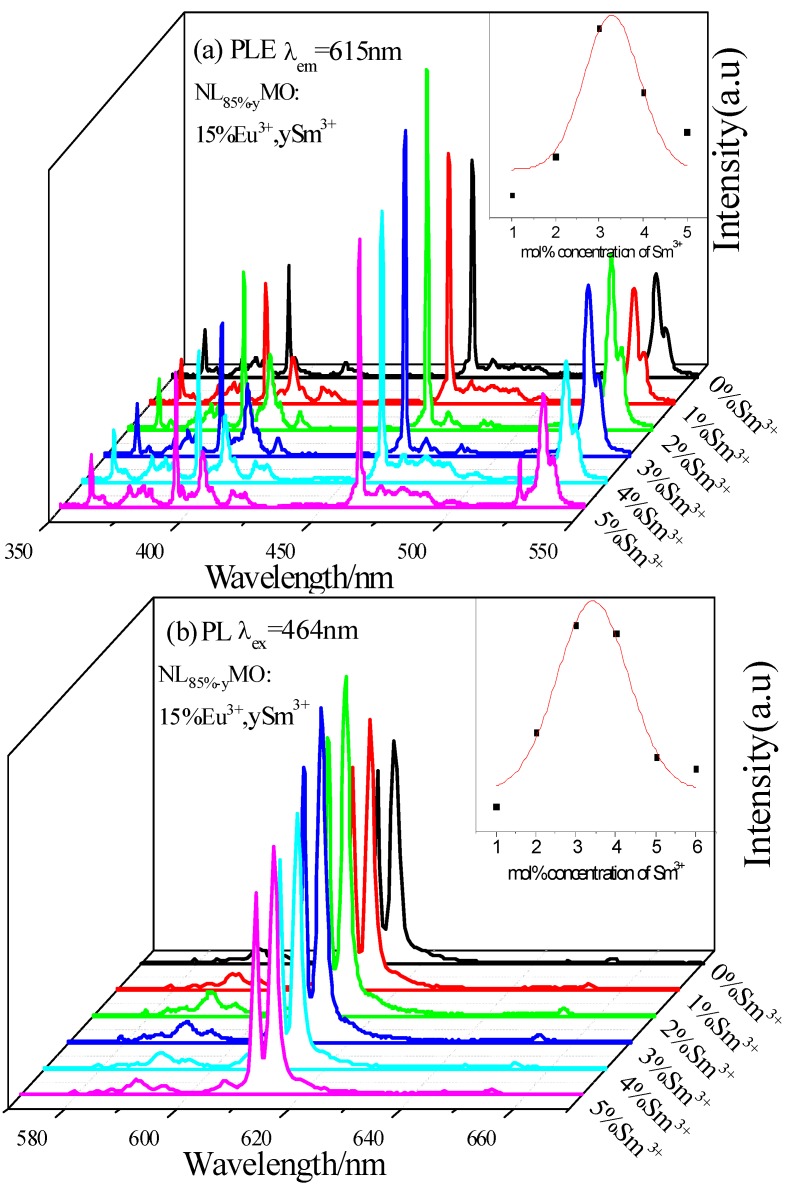
(**a**,**b**) excitation spectra (λ_em_ = 615 nm) and emission spectra (λ_ex_ = 464 nm) of NL_85%-y_MO:15%Eu^3+^,ySm^3+^ (y = 0, 1, 2, 3, 4 and 5%).

Figure 8a,b exhibits the PLE (λ_em_ = 615 nm) and PL(λ_ex_ = 464 nm) spectra of NL_85%−y_MO:15%Eu^3+^,ySm^3+^ (y = 0, 1, 2, 3, 4 and 5%), respectively. Clearly, with y increasing from 0 to 2%, the characteristic excitation and emission intensities of Eu^3+^ are enhanced because Sm^3+^ transfer its energy to Eu^3+^, and, then, the intensities are weakened because of concentration quenching. Moreover, the fluorescence intensity of NL_83%_MO:15%Eu^3+^,2%Sm^3+^ is 1.47 times that of NL_83%_MO:15%Eu^3+^. The results further demonstrate the efficient energy transfer from Sm^3+^ to Eu^3+^.

### 3.4. Decay Curves and Energy Transfer Mechanism of NLMO:Eu^3+^/Sm^3+^ Phosphors

**Figure 9:**
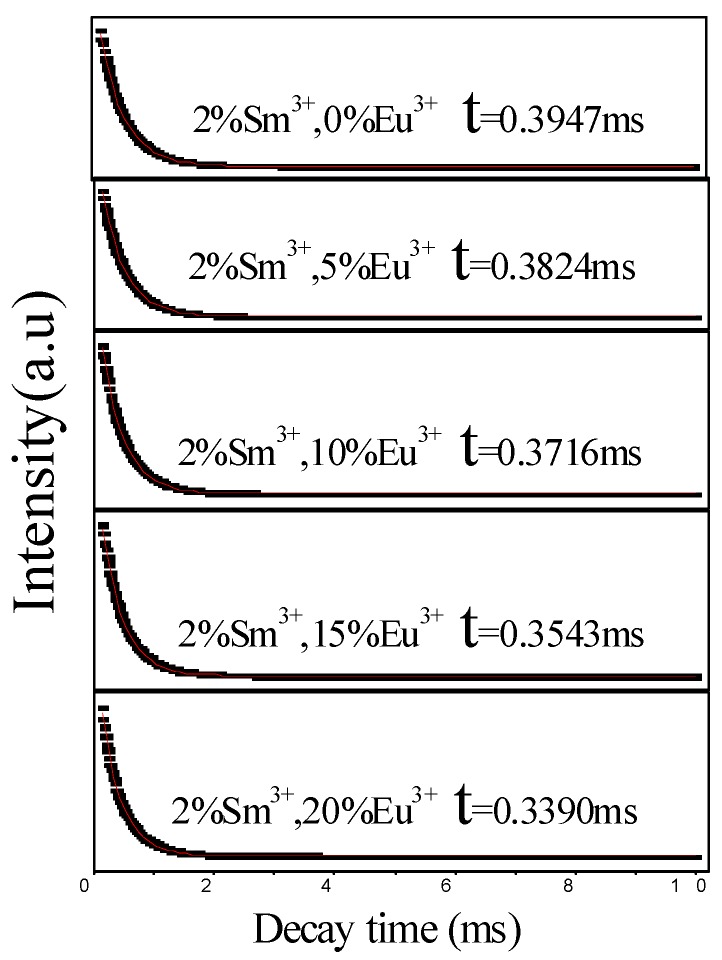
PL decay curves of NL_98%-x_MO:xEu^3+^,2%Sm^3+^.

To deeply explore the interaction mechanism of energy transfer from Sm^3+^ to Eu^3+^ in the NLMO matrix, we measured the fluorescence lifetime of NL_98%−x_MO:xEu^3+^,2%Sm^3+^ (x = 0, 5, 10, 15 and 20%). The test results and decay curves were shown in Table 1 and Figure 9, respectively.

The fluorescence lifetime of Sm^3+^ is gradually shortened along with the concentration of Eu^3+^ increased from 0 to 20%, which is due to the fact that the energy transfer between Sm^3+^ and Eu^3+^ shortens the time for excited-electrons to move back to the ground state. To more intuitively understand the energy transfer from Sm^3+^ to Eu^3+^, the efficiency (η_ET_) of energy transfer from Sm^3+^ to Eu^3+^ was calculated using the following formula [24,25] and the results are also listed in Table 1:

(1) $$\eta_{ET}=1-\frac{\tau_{s}}{\tau_{s0}},$$

where τ_s0_ and τ_s_ are the lifetime durations of the donor (Sm^3+^) without and with the acceptor (Eu^3+^), respectively. All of the above results strongly confirm that Sm^3+^ transfers its excitation energy to Eu^3+^ in the NaLa (MoO_4_)_2_ matrix.

**Figure 10:**
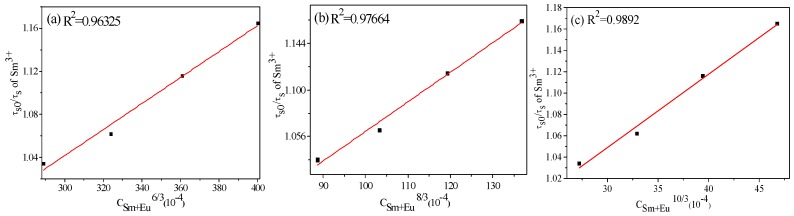
Dependence of τ_s0_/τ_s_ on C^α/3^ with α = (**a**) 6, (**b**) 8, (**c**) 10.

Energy is transferred through primarily exchange interaction and multipolar interaction, when the distance, R, between the donor ion and the acceptor ion is less than 0.5 nm and more than 0.5 nm, respectively [26,27]. R_Sm−Eu_ between Eu^3+^ and Sm^3+^ in the NaLa(MoO_4_)_2_ matrix can be calculated as follows [28] (Table 1):

(2) $$R_{\mathrm{Sm}-\mathrm{Eu}}=2(\frac{3V}{4\pi \chi_{C}N}{)}^{1/3},$$

where V is the unit cell volume, *χ*_c_ is the total doping mol % concentration of Sm^3+^ and Eu^3+^, and *N* is the number of cation sites. For the NaLa(MoO_4_)_2_ crystals, V = 0.33501 nm^3^ and *N* = 2. Based on the above data, the R_Sm−Eu_ of NL_98%−x_MO:xEu^3+^,2%Sm^3+^ (x = 0%, 5%, 10%, 15% and 20%) can be calculated (Table 1). Obviously, the R_Sm−Eu_ values are much greater than 0.5 nm, indicating the exchange interaction is greatly limited. Therefore, Sm^3+^ transfers its energy to Eu^3+^ through the multi-pole interaction mechanism. According to the Dexter formula [29] and Reisfeld approximation method [30] of the electric multi-pole interaction mechanism, the following relation can be obtained:

(3) $$\frac{\tau_{s}}{\tau_{s0}}\propto C^{\alpha /3},$$

where C is the doping mol % concentration of Sm^3+^ and Eu^3+^, and α is relationship index. The values of α correspondent to dipole–dipole, dipole–quadrupole and quadrupole–quadrupole interactions are 6, 8 and 10, respectively. Figure 10a–c shows the Gauss plot of the linear dependency between τ_s0_/τ_s_ and C^α/3^. Clearly, R^2^, the goodness of fit, is closer to 1 at α = 10, meaning Sm^3+^ transfers its energy to Eu^3+^ in the NaLa(MoO_4_)_2_ matrix by the mechanism of quadrupole–quadrupole interaction.

### 3.5. Chromaticity Coordinate Analysis

**Figure 11:**
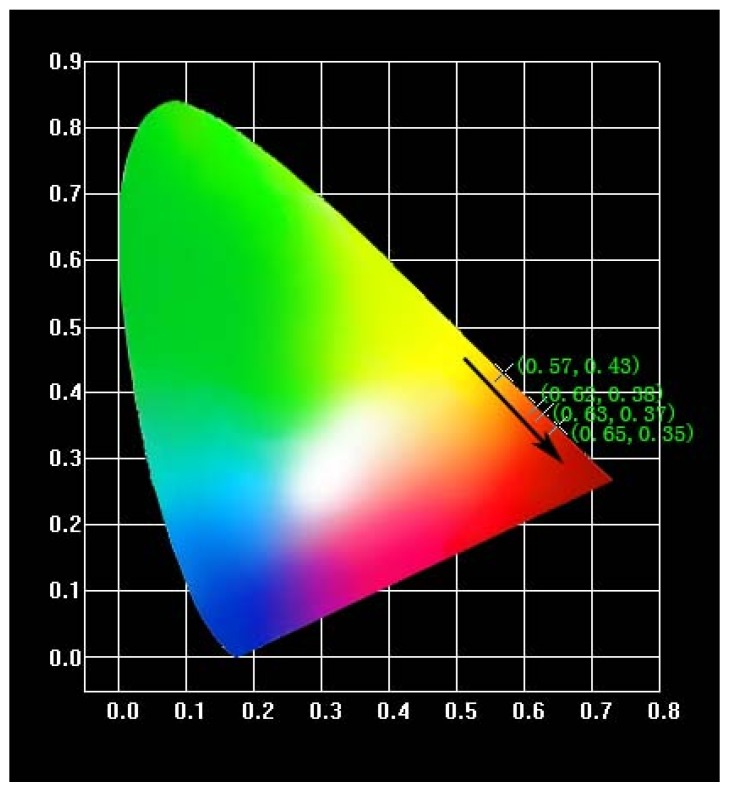
CIE chromaticity diagram of NL_85%−y_MO:15%Eu^3+^,ySm^3+^ phosphors excited at 464 nm.

To investigate the luminous color of the phosphors, we depicted the CIE chromaticity diagrams of NL_85%-y_MO:15%Eu^3+^,ySm^3+^ when they are excited at 464 nm (Figure 11). The CIE coordinates are listed in Table 2. As seen, the emission light can be regularly shifted from orange to red with the Sm^3+^ molar ratio increasing with a maximum at (0.65, 0.35), which is very close to the CIE chromaticity coordinates (0.67, 0.33) of the standard red light, indicating NLMO:Eu^3+^,Sm^3+^ has a potential application prospect in blue-light-excited WLEDs.

## 4. Conclusions

We prepared the NLMO:Eu^3+^/Sm^3+^ phosphors via solid-station reaction at 450–600 °C and analyzed their fluorescence spectra. The X-ray diffraction analysis showed that the phosphors possess a body-centered tetragonal structure with high purity and crystallinity. The fluorescence spectrum observation indicates that the phosphor has the strongest excitation and emission peaks at 464 and 615 nm, respectively. The composition possesses a potential for WLEDs because of efficiently emitting red when excited by blue light. In-depth analysis of fluorescence spectra, fluorescence lifetime and inter-ion distance reveals that the energy transfer from the ^4^G_5/2_ state of Sm^3+^ to the ^5^D_0_ state of Eu^3+^ is achieved via the quadrupole–quadrupole mechanism, and it finally broadened the absorption band of Eu^3+^ and enhanced the luminous intensity.

## Figures and Tables

**Table 1:** Energy transfer efficiency, distance, lifetime of NL_98%-x_MO:xEu^3+^,2%Sm^3+^.

Xc/mol %	η_ET_/%	R_Sm−Eu_/nm	τ_s_/ms
2	-	2.5196	0.3947
7	3.116	1.6595	0.3824
12	5.853	1.3866	0.3716
17	10.236	1.2346	0.3543
22	14.112	1.1329	0.3390

**Table 2:** CIE chromaticity coordinates (x, y) of NL_85%-y_MO:15%Eu^3+^,ySm^3+^ (y = 0, 1, 2, 3, 4 and 5%) excited at 464 nm.

NL_85%-y_MO:15%Eu^3+^,ySm^3+^/%	CIE x	CIE y
y = 0	0.57	0.43
1	0.62	0.38
2	0.63	0.37
3	0.65	0.35
4	0.65	0.35
5	0.65	0.35
